# Mental health challenges among adolescents living with HIV

**DOI:** 10.7448/IAS.20.4.21497

**Published:** 2017-05-16

**Authors:** Rachel C. Vreeman, Brittany M. McCoy, Sonia Lee

**Affiliations:** ^a^ Indiana University School Medicine, Department of Pediatrics, Indianapolis, Indiana, USA; ^b^ Academic Model Providing Access to Healthcare (AMPATH), Eldoret, Kenya; ^c^ Moi University, College of Health Sciences, School of Medicine, Department of Child Health and Paediatrics, Eldoret, Kenya; ^d^ National Institutes of Health, Eunice Kennedy Shriver National Institute of Child Health and Human Development, Maternal and Pediatric Infectious Disease Branch, Bethesda, Maryland, USA

**Keywords:** Adolescents, HIV, mental health, transition, stigma, mental health disorders

## Abstract

**Introduction**: Mental health is a critical and neglected global health challenge for adolescents infected with HIV. The prevalence of mental and behavioural health issues among HIV-infected adolescents may not be well understood or addressed as the world scales up HIV prevention and treatment for adolescents. The objective of this narrative review is to assess the current literature related to mental health challenges faced by adolescents living with HIV, including access to mental health services, the role of mental health challenges during transition from paediatric to adult care services and responsibilities, and the impact of mental health interventions.

**Methods**: For each of the topics included in this review, individual searches were run using Medline and PubMed, accompanied by scans of bibliographies of relevant articles. The topics on which searches were conducted for HIV-infected adolescents include depression and anxiety, transition from paediatric to adult HIV care and its impact on adherence and mental health, HIV-related, mental health services and interventions, and the measurement of mental health problems. Articles were included if the focus was consistent with one of the identified topics, involved HIV-infected adolescents, and was published in English.

**Results and Discussion**: Mental and behavioural health challenges are prevalent in HIV-infected adolescents, including in resource-limited settings where most of them live, and they impact all aspects of HIV prevention and treatment. Too little has been done to measure the impact of mental health challenges for adolescents living with HIV, to evaluate interventions to best sustain or improve the mental health of this population, or to create healthcare systems with personnel or resources to promote mental health.

**Conclusions**: Mental health issues should be addressed proactively during adolescence for all HIV-infected youth. In addition, care systems need to pay greater attention to how mental health support is integrated into the care management for HIV, particularly throughout lifespan changes from childhood to adolescence to adulthood. The lack of research and support for mental health needs in resource-limited settings presents an enormous burden for which cost-effective solutions are urgently needed.

## Introduction

### Mental health issues and adolescents with HIV

Mental health is a neglected global health priority, particularly for children and adolescents [1–3]. Mental health disorders, including psychiatric disorders, general psychological distress, emotional, and behavioural problems, are a leading cause of health-related disability, affecting 10–20% of children worldwide [4], and are predictive of mental health disorders and other morbidities in adulthood [5,6]. Research on mental health among children and adolescents lags considerably behind that of adults, particularly in resource-limited settings (RLS) [7,8]. A 2007 review of 11,501 intervention trials for the treatment or prevention of mental health disorders found that few targeted children and adolescents; the little research with children primarily focused on interventions for developmental disabilities [9]. Furthermore, less than 1% of the studies of drug and psychological interventions for treating mental health disorders were conducted in “low-income” countries, with only 10% in “lower-middle-income” countries [9].

The need for a better understanding of mental health is especially important when its assessment and treatment are compounded by other comorbidities. Children and adolescents living with HIV may face an increased burden of mental and behavioural health disorders. The objective of this narrative review is to assess the current literature related to mental health challenges faced by adolescents living with HIV, including access to mental health services, the role of mental health challenges during transition from paediatric to adult care services and responsibilities, and the impact of mental health interventions. This review focuses on adolescents who are themselves infected with HIV, with particular attention to: (1) the developmental phase of adolescence, and (2) to the specific challenge of transitioning primary responsibility of their medical care.

## Methods

Individual searches were run using Medline and PubMed for each of the topics included in this review. The topics on which searches were conducted included the following: (1) depression and anxiety among HIV-infected adolescents, (2) transition from paediatric to adult HIV care and its impact on adherence and mental health, (3) HIV-related stigma and mental health, (4) mental health services for adolescents in RLS and interventions for HIV-infected adolescents, and (5) the measurement of mental health problems in adolescents with HIV.

Exact text and MeSH headings for search terms are available from the authors on request (also see Table 1.). Articles on measuring mental health problems in adolescents with HIV were taken from the preliminary results of an ongoing systematic review on the use of mental health screening instruments among HIV-infected, paediatric and adolescent populations in Africa.

**Table 1.** Search terms to identify articles.

Main topic	Specific search terms	Additional inclusion criteria/Steps
Depression and anxiety	*depression, mood disorders, depressive disorder, anxiety, anxiety disorders, HIV, HIV infections, adolescent*	
Transitioning to adult HIV care	*transition, transition to adult care, HIV, HIV infections*	Addressed mental health impact or impact on adherence to antiretroviral medication regimens
HIV stigma	*HIV, stigma, mental health, adolescents*	
Mental health services for adolescents in resource-limited settings	*mental health services, adolescent, resource-limited, low- and middle-income country*	
Mental health interventions for HIV-infected adolescents	*HIV, HIV infection, adolescent, mental health, intervention*	
HIV-infected adolescents	*HIV, HIV infections, depression, mood disorders, depressive disorder, anxiety, anxiety disorders, validation studies, reproducibility of results, predictive value of tests, sensitivity and specificity, psychometrics, mass screening, surveys and questionnaires, Africa*	Searched PsychINFO, EMBASE, Medline; Reviewed titles, followed by Abstract review

Articles from these searches were included if the focus of the study or review was consistent with one of the identified topics, the study or studies reviewed involved adolescents, and the article was published in English. We then identified additional articles meeting our inclusion criteria from the bibliographies of articles identified in our searches, as well as by using the “cited by” function through PubMed. From each article identified, we extracted information on the study design, population, geographical context, measurement methods, and study outcomes from each study.

## Results and discussion

### Prevalence of mental health challenges in HIV-infected children and adolescents: high-income countries

Studies from high-income settings generally suggest that children and adolescents with HIV face an increased burden of mental health challenges. HIV-infected children and youth are at higher risk of psychiatric hospitalizations, compared with the general paediatric population [10]. A 2013 systematic review of the literature on the mental health of adolescents living with HIV found few studies describing the prevalence of psychiatric diagnoses in HIV-infected adolescents, but the existing studies suggest that psychiatric disorders such as depression and anxiety are more prevalent among perinatally infected adolescents compared to non-infected adolescents [11]. With the advent of antiretroviral therapy (ART), despite a significant decline in the incidence of severe, AIDS-defining neurocognitive diseases like HIV encephalopathy [12,13], HIV-infected children may still experience neurocognitive complications, such as deficits in cognitive, speech, gross motor and fine motor functioning, that can substantially impact their quality of life (QoL), social relationships, academic achievements, and risks for abuse and substance use [14–18]. These complications can be seen despite early ART treatment and viral suppression [19]. Moreover, HIV-infected adolescents are growing up and developing in a milieu that includes exposure to biomedical, genetic, familial, economic, and social or environmental factors that may increase their risk for mental health problems [20].

An older review of the prevalence of DSM psychiatric disorders among HIV-infected children and adolescents found prevalence of 29% for Attention Deficit Disorder, 24% for anxiety disorders, and 25% for depression; however, appropriate control groups were infrequently used [21]. In a large longitudinal cohort study of youth in New York who were perinatally exposed to HIV, 61% were found to have psychiatric disorders other than substance use on the Diagnostic Interview Schedule for Children (DISC-IV), but only the prevalence of ADHD was different between the HIV-infected and HIV-exposed groups [22]. Prevalences of anxiety, mood, and other behavioural disorders were the same between the HIV-exposed groups, with no inclusion of a non-HIV-exposed comparison group. A 2000 study in the United States found that among HIV-positive adolescents, 53% had received psychiatric diagnoses prior to HIV treatment, and 44% experienced ongoing depressive disorders [23].

Many of the studies of mental health in HIV-infected children and adolescents conducted to date lack comparison groups, which makes it difficult to compare the prevalence of mental health challenges in this population to the general population or to draw conclusions about the potential contribution of HIV on the observed rates of mental health challenges [11]. Due to the complexity of the effects of HIV, appropriate comparison groups could include adolescents who were perinatally exposed to HIV, but remain HIV uninfected, as well as adolescents without any history of HIV exposure, but from similar communities and backgrounds. Available studies that compare perinatally infected, HIV-positive adolescents with those perinatally exposed to HIV, but uninfected largely suggest that there are not significant differences in the rates or types of psychiatric disorders between these groups in comparison with those with no HIV exposure [11,22,24,25].

### Prevalence of mental health challenges in HIV-infected children and adolescents: low- and middle-income countries

Most studies evaluating the prevalence of mental health challenges for HIV-infected adolescents have been done in the United States, rather than in the RLS where most of the world’s HIV-infected adolescents live [11]. Fewer data are available on mental health among HIV-infected adolescents in RLS [11,26], which may be partially related to a lack of validated instruments for these contexts, lack of attention to or resources for mental health in resource-constrained healthcare settings, and additional stigma related to mental health conditions [27]. Despite these challenges, accumulating evidence from RLS does suggest that adolescents living with HIV also face particular emotional, behavioural, and mental health challenges.

Most studies of mental health challenges among adolescents living with HIV in low- and middle-income countries are cross-sectional and do not include comparison groups; however, they do indicate the need to address mental health within care systems addressing HIV or primary care. In a study of 162 HIV-infected children and adolescents in Kenya, 49% were reported to have at least one psychiatric diagnosis or suicidality, with anxiety disorders most common (32.3%), followed by major depressive disorder (17.8%) [28]. A cross-sectional study of 562 HIV-infected adolescents from Malawi found a depression prevalence of 18.9% [29,30]. Within another study in Rwanda examining 100 HIV-infected children ages 7–14 years, the prevalence of depression reported was 25% [31]. A cross-sectional study of 82 HIV-infected adolescents ages 10–18-years old in Kampala, Uganda found that 51.2% had scores indicating significant psychological distress, 17.1% had attempted suicide in the past year, 19.5% had ever attempted suicide, and 30.5% had experienced psychotic symptoms in the past [32].

A cross-sectional study of 692 HIV infected, treated children ages 8–17 years in Botswana using a culturally-adapted and translated version of the Pediatric Symptom Checklist found that higher scores on the PSC (indicating psychosocial dysfunction) were associated with virologic failure, suggesting a critical link between psychosocial function and clinical outcomes [33]. In Tanzania, a cross-sectional study of 182 HIV-infected adolescents between 12 and 24 years old found multiple suggestions of mental health challenges in this group [34].

The few studies that use comparison groups in these settings do suggest certain increased risks of mental health challenges for those living with HIV. A study of 683 children ages 10–17 years of age in Rwanda compared a group with either HIV infection themselves (HIV-infected) or who were HIV affected (living with an HIV-infected caregiver or had a caregiver who had died from HIV) to a control group that was neither HIV infected nor living with anyone HIV infected [35]. Twenty per cent of the HIV-infected or HIV-affected adolescents were reported to have attempted suicide or engaged in self-harm in the past 6 months, compared to 13% of HIV-uninfected, unaffected children, with child-reported HIV-related stigma significantly increasing the risk of suicidal ideation and behaviours [27]. The HIV-infected or affected group of children also had significantly increased risks of depression, anxiety, and conduct problems compared to the HIV-uninfected/HIV-unaffected adolescents, but this did not differ whether the youth were themselves infected or had a HIV-infected caregiver or caregiver who had died from HIV [26]. In another study comparing HIV-positive Zambian adolescents with a control group from a British community sample, the HIV-infected Zambian adolescents had significantly higher reports of emotional symptoms, hyperactivity, and peer problems [36]. These combined studies suggest that, while HIV-infected adolescents in RLS are also at risk of developing mental health problems such as depression, anxiety, and ADHD, the aetiology extends beyond their HIV infection or health status and is likely impacted by the full range of their biopsychosocial experiences.

### Measurement of mental health among adolescents: high-income settings

Measuring mental and behavioural health for adolescents remains a critical challenge that must be addressed in order for mental health to be supported and improved. A 2015 systematic review examined depression screening instruments used to detect major depressive disorder in children and adolescents generally, for those ages 5–18 years [37]. The vast majority of studies evaluating the reliability and validity of depression measurement for children and adolescents have been conducted in the United States and Europe [37]. Four screening instruments, the Children’s Depression Inventory (CDI), the Beck Depression Inventory (BDI), the Center for Epidemiological Studies – Depression Scale (CES-D), and the Reynold’s Adolescent Depression Scale (RAD), were identified as the most commonly used tools and all were found to be reliable measures of Major Depressive Disorder in children and adolescents [37]. These four screening instruments are also commonly used to assess depression among HIV-infected adolescents in high-income countries. Though more often used with adults, the Patient Health Questionnaire (PHQ)-9 is a brief, well-validated screening measure that is available in more than 60 languages [38].

### Measurement of mental health among adolescents: low- and middle-income settings

Few instruments to evaluate mental and behavioural health outcomes have undergone rigorous evaluation and validation for adolescents in RLS [39]. Research in these settings typically employs instruments and criteria developed in high-income settings that, if culturally inappropriate in a certain context, could lead to erroneous or misleading results [40,41]. The appropriateness of concepts and instruments may vary across different cultures [42] and across populations such as children and adolescents [43]. Betancourt *et al*. conducted a validation study to test the validity of the CES-DC as a depression screen for a general population sample of 367 children and adolescents in Rwanda, which included translation and cognitive testing of the screening items [44]. They found the CES-DC to be a valid screening tool, with a sensitivity of 81.9% and specificity of 71.9%, and made a recommendation that similar research efforts validate measures according to how well the measure items fit with local characterizations of mental health problems [44,45].

There is even more limited evidence for how to examine mental health outcomes among HIV-infected adolescents in RLS. Kim *et al*., Mutumba *et al*., and Binagwaho *et al*.tested the validity of these existing mental health measurement instruments within Malawi, Uganda, and Rwande, respectively [29,31,46]. In Malawi, the validation findings suggested that the BDI-II had greater internal consistency and showed greater concordance with the CDRS-R results for this population [29]. The studies from Uganda and Rwanda highlighted the need for a process of cultural adaptation, such as included translation, expert panel review, cognitive interviewing, and pilot testing, followed by full evaluation to modify these instruments and generate sufficient sensitivity for use [31,46]. These studies highlight the importance of cross-cultural modification of instruments and local validation, while also demonstrating the need to employ rigorous measurement of mental health symptoms for HIV-infected populations of children and adolescents in the RLS where most of these children live.

### Mental health and sex-based differences across income settings

Sex-based differences in mental health challenges among HIV-infected youth have not been consistently identified although there seems to be a suggestion that females are at higher risk [47,48]. As Mellins *et al*. noted in their 2013 systematic review, some studies have found that, among HIV-infected youth, female sex is a risk factor for depression and anxiety, while male sex is a risk factor for behavioural problems [11]; however, other studies have had mixed or inconsistent results regarding gender differences.

There is more variation in the findings related to biological sex among HIV-infected adolescent in low-income settings. One study found male sex to be associated with a greater risk of depression than female sex in Kenya [28], while another found female sex to be associated with higher BDI-II scores in Malawi [30]. Another study found depression rates to be higher among HIV-infected adolescent females than males in Rwanda, but this difference was not significant [31], while another found no significant association between sex and suicidal ideation or behaviour in Rwanda [35].

### Mental health associated with adolescents’ ART adherence across income settings

Adherence to ART is critical to maintaining viral suppression and avoiding morbidity and mortality among HIV-infected patients. Among HIV-infected children and adolescents, depression and anxiety symptoms have been associated with lower adherence to ART [49–51] and higher substance abuse and risky sexual behaviours [52]. In a longitudinal study of 294 perinatally HIV-infected children and adolescents in the US and Puerto Rico, children with anxiety were 40% less likely to have unsuppressed viral load compared to other children [53]. Mental health disorders may be exacerbated by social exclusion and HIV stigma, which are associated with delayed HIV testing [54,55] and decreased treatment adherence [56]. Stigmatizing aspects of HIV infection or treatment, such as lipodystrophy, may also negatively impact mental health outcomes like depression and adherence to ART among adolescents, particularly during this developmental period when body image and the social desirability to “fit in” are strong motivators of behaviour [57]. Behaviourally HIV-infected youth who experience a combination of individual challenges such as low levels of self-efficacy and mental health disorders, in combination with environmental challenges such as homelessness or a history of time in detention facilities, have greater issues with maintaining adherence [58,59].

Based on a qualitative study of perinatally infected adolescents in Canada, how medication adherence is interpreted may be the key to whether the adolescent begins to take responsibility for his/her own HIV care, including adherence [60]. A positive mental framework towards adherence may be associated with autonomy and control over the adolescent’s health and wellbeing. Conversely, a negative mental framework views adherence as a reminder of HIV infection, difference from peers, and stigmatization. This interplay of mental health, adherence, and the adolescents’ psychosocial environment suggests a critical role for mediating these risk factors and boosting mental and emotional health resilience for these adolescents.

### HIV-related stigma and adolescent mental health across income settings

HIV-related stigma is a key issue that impacts adolescents living with HIV across country-income settings by affecting quality of life, healthcare access, and health outcomes. Stigma and discrimination experienced by HIV-infected youth through the broader community, as well as in clinical encounters, are significant barriers to HIV treatment, often leading to negative consequences and poor health outcomes [34,61]. Furthermore, HIV-related stigma is often intertwined with other sources of stigma, including those associated with mental health and/or substance use disorders. Research that investigates these impacts upon public health can guide the development of service delivery and provision of optimal healthcare appropriate for the resource setting. Such interventions to combat barriers due to stigma are especially relevant for adolescents transitioning their medical care to adult care settings, as the burden and interplay of physical, emotional, and social stressors during this vulnerable, developmental period increase. For example, due to the expectations of increased independence in navigating the healthcare systems, adolescents with HIV may need increased social support from family and friends. However, the availability and level of social support for adolescents and young adults living with HIV may be complicated by mental health issues, many of which may be emerging during this time, as well as stigma and disclosure [62]. Services to address stigma, social support, and mental health overall, are scarce, especially in low-resource settings [62,63]. Additional evidence on the impact of stigma, HIV related, as well as mental health related, on the transition process for adolescent to adult care settings and resultant health outcomes is needed.

### Mental health within the transition from paediatric to adult medical care: high-income settings

Adolescents’ mental health must be considered throughout the process of transitioning to adulthood with HIV, both in the general sense of their transition to adult, autonomous management of their disease as well as specific changes that may occur in the provision of their medical care. One of the first steps in adolescents’ transition to managing their own HIV care is being informed of their own HIV status, also referred to as child HIV disclosure. Decisions around when to disclose HIV status to children are often impacted by caregivers’ beliefs about the impact of disclosure on the child’s mental health [64]. Studies suggest that disclosure of their own HIV status to children causes stress [65], but whether the disclosure process has a lasting negative impact on mental health is not clear [36,66]. In settings with higher HIV-related social exclusion and stigma, disclosure of HIV status to adolescents is also likely to be delayed [67].

In addition to this period of developmental transition from childhood to adulthood, adolescents may experience a physical transition in who provides their HIV care or where they receive medical services. Transitions that include an entirely new environment in which to receive medical care, potentially with fewer psychosocial supports, a new set of providers, or less “protective” HIV care compared to child-centred programmes, may create feelings of fear and anxiety for adolescents, even among those without any pre-existing mental health conditions [68]. In qualitative work, adolescents report feeling unprepared for transition and anxious about transitioning [69,70]. In addition to adjusting to new providers and care settings, the mental and emotional stressors may include the stress of disclosing their HIV status to providers within the new care system, the possibility of facing new HIV-related stigma or discrimination, and retelling their often-traumatic life story once again [65]. In addition, the new care systems in which adolescents find themselves may not provide care tailored to adolescents’ specific needs, which may include sexual and reproductive healthcare, addressing specific populations such as young men who have sex with men, and adolescents’ concerns about disclosing either their HIV status or sexual health issues to family members [65].

Mental health challenges likely complicate the navigation of these changes in medical care. In a systematic review of studies of HIV-infected youth transitions into adult care, the combined barriers to transition anticipated by providers and the adolescents included feelings of loss when separating from the paediatric provider, anxiety about increasing autonomy, and the logistic challenges of navigating the adult healthcare system – all of which may colour the mental and emotional health state of the adolescents [71]. A majority of providers of adolescent HIV care surveyed in the United States expressed concern that those adolescents who do have mental health problems or substance use issues would be at risk for being lost to care in the process of transition, especially as the adult care services might not follow the same multidisciplinary and integrated care models often seen in the paediatric HIV care models in this setting [72]. The preparedness of adult providers to address a population with higher rates of cognitive impairment, mental health problems, and adherence difficulties may be a challenge throughout the healthcare transition [65]. In a qualitative study of American HIV care providers, the following indicators of successful transition to adult care were identified: medication adherence, adherence to clinic visits, taking ownership of medical care, viral load, and CD4 count [73]. Adolescents with mental health problems or struggling with the multiple relational and environmental factors that make them more prone to mental health symptoms may be less likely to have these characteristics. In another qualitative study of clinical and programme staff providing adolescent HIV care at 12 clinical sites across the United States, the identified barriers to successful transition included mental health issues, substance abuse, paediatric providers being hesitant to transition care, and issues with insurance coverage [74].

### Mental health within the transition from paediatric to adult medical care: low- and middle-income settings

Very few studies have examined the barriers and facilitators of transition in RLS, with even less data regarding potential mental health challenges. In many RLS, particularly in sub-Saharan Africa, there are not separate paediatric specialty services for HIV-infected children, nor is there specialized care for adolescents (with or without HIV), and so HIV-infected adolescents may not experience any changes in providers or care systems in these settings. One qualitative study in Thailand, which employed semi-structured interviews of users of ART services, policymakers, and caregivers of orphaned children, concluded that adherence, drug resistance, and psychosocial issues were important in caring for HIV-infected children long term, that services to help adolescents to transition into adult care were largely lacking, and that physicians did not have adequate skills or comfort to aid in this transition. However, mental health needs or issues were not considered in depth [75].

### Mental health service access for adolescents: high-income countries

Considering how to ensure access to resources for mental health services for HIV-infected adolescents is a critical consideration [69]. A 2011 study used the World Health Organization Assessment Instrument for Mental Health Systems (WHO-AIM) in 13 low-, 24 lower-middle, and 5 upper-middle income countries. One indicator from this instrument included the treated child and adolescent prevalence in all mental health facilities for a one-year period. It found a one-year median treated prevalence for children and adolescents of 159 per 100,000 patients treated compared to 664 per 100,000 in adults, with greater differences in the low- and lower-middle-income countries than upper-middle-income countries [76]. Children were more likely to be treated in outpatient facilities and made up 12% of the mental health outpatient population, but none of the other facilities surveyed (e.g. inpatient facilities, day treatment) had any facilities devoted to children [76]. Even in resource-rich settings, there may be important disparities in mental health service access for minority populations; in the U.S. Adolescent Treatment Network, Black HIV-infected youth were less likely to receive care for mental health symptoms than non-Black youth [77]. Among 164 HIV-infected adolescents in 3 U.S. cities enrolled in the Adolescent Impact study, 31% had symptoms of psychopathology, but almost one-third of those reporting clinically significant symptoms did not receive care despite the availability of psychiatric medications, hospitalizations, counselling, or psychotherapy [78].

### Mental health service access for adolescents: low- and middle-income countries

Mental health services for children and adolescents in resource-limited countries are extremely limited according to existing estimates, and thus, lead to severe limitations in not only access to, but also uptake of mental health services [76]. The availability of providers may be extremely limited; only 1% of schools in low- and middle-income countries had mental health professionals as staff members, and less than 1% of professionals had attended any training on child and adolescent psychiatry in the last year [76]. Ratios of available psychiatrists are estimated at 1 psychiatrist per 4 to 5 million children in resource-limited countries, and training for mental health workers are limited and often out-of-date [79]. Qualitative inquiry among key education personnel and teachers in Nigeria suggests that, in some settings, these deficits in services may be further hampered by stigmatizing beliefs about mental health disorders, disbelief that children can have a mental illness, and overtly discriminating or derogatory language and treatment used in regards to mental illness [80]. Access to mental health services in low- and middle-income countries may also be affected by concerns regarding the use of psychotropic medications in conjunction with ART. Options to extend mental health services through alternative modes of delivery such as teleconsultations and telepsychiatry may offer particular benefits for RLS with adolescents in need of mental health screening and treatment [79]. An integrated mental healthcare approach within the primary HIV care setting to address gaps in mental health service access and treatment is another option worth investigating [81].

### Mental health interventions for HIV-infected adolescents: high-income countries

Few mental health interventions, specifically for HIV-infected adolescents, have been studied. One pilot study from a resource-rich setting used health and wellness cognitive behavioural therapy for 8 HIV-infected participants aged 16–24 years and found significant improvement in depression symptoms as measured over 14 sessions by the Quick Inventory of Depressive Symptomatology-Clinician measure, as well as improved self-reported adherence [82]. In a literature review of studies evaluating the impact of service delivery interventions to improve the health of perinatally HIV-infected adolescents, the overall conclusion from the 12 studies examined was that youth-focused health services and individual-level interventions would improve adherence and retention in care; however, these were small studies with limited follow-up times, and the impact of the service delivery interventions on mental health were largely unexamined [83].

### Mental health interventions for HIV-infected adolescents: low- and middle-income countries

Interventions to promote mental health show significant promise generally to improve child and adolescent mental health and wellbeing in RLS. A systematic review of mental health interventions and their effectiveness for adolescents in resource-limited studies identified 14 studies of school-based interventions and 8 studies of community-based interventions [84]. The majority of the school-based interventions led to improvements in student emotional and behavioural wellbeing, self-esteem, and coping skills [84]. Similarly, community-based interventions also showed significant improvements in youth mental health and social wellbeing [84]. Cognitive-behavioural group therapy has been shown to improve externalizing symptoms and anxiety in low- and middle-income countries, though not depressive symptoms or quality of life [85]. Very few mental health interventions have been studied specifically for HIV-infected adolescents in RLS.

## Conclusions

This review of the literature related to mental health, HIV-infected adolescents, and transition raises the critical importance of considering mental health as an integral component to adolescent care and to the transition process. While mental health disorders are often prevalent in this population, too little has been done to measure the impact of these disorders for adolescents, to evaluate interventions to best sustain or improve the mental health of this population, or to create healthcare systems with personnel or resources to promote mental health. Evidence-based studies confirming the link between poor mental health and deleterious health outcomes are still needed within the growing population of HIV-infected adolescents to inform guidelines and policies for HIV healthcare.

For the low- and middle-income countries in which the majority of the world’s HIV-infected adolescents live – and the exact settings in which the rates of adolescent HIV-related deaths are a major concern – the absence of screening for mental health disorders, the lack of evidence for how to intervene to prevent or improve mental health problems, and the minimal healthcare infrastructure to address mental health are enormous obstacles. These limitations for mental health are all too-often complicated by HIV-related stigma, mental health-related stigma, sex and gender disparities, and the broader set of psychosocial challenges faced by adolescents living with HIV. Any effort to provide long-term care for these youth and to effectively support their transition into adulthood must address the adolescents’ mental health. Validating mental health screening tools that can be used in routine care settings in RLS should be a priority research area.

Mental health is also likely critical for adolescents’ successful transitions within the specific realm of medical care services as they face the long-term challenges of remaining in medical care, sustaining medication adherence, and achieving viral suppression. Further research to assess these connections and how to sustain them is urgently needed for this population, particularly in low- and middle-income countries. For transition processes to meet the full range of adolescents’ mental health and emotional health needs, transition plans need to consider HIV-infected adolescents’ risk of mental health disorders and psychiatric illness, the specific needs of adolescents, and the key issues of HIV-related stigma, HIV disclosure to others, adherence issues, and the range of factors that may increase or decrease resilience in the face of transitions in care [69]. For example, detailed recommendations for the medical transition process from the Johns Hopkins University HIV Clinical Guidelines Program (2000–2016) in the United States emphasize the need for creating a plan for each adolescent that does the following: (1) “Address the individual barriers for each patient that may be preventing him/her from acquiring skills, such as anxiety, depression” and (2) Provide HIV care “in settings where patients can receive all services in one location from a multidisciplinary team. If a multidisciplinary team is not available, mental health and psychosocial support services should be available onsite or in an easily accessible location” [86]. A multidisciplinary team approach to the screening of mental health disorders, followed by an integrated treatment approach with primary HIV care along the transition continuum could improve access to and uptake of mental health services and interventions.

This review offers an update on previous systematic reviews of mental health challenges and interventions, which have focused on the more general population of children and adolescents [4,7,8], by assessing emerging work of those infected with HIV from RLS [11]. While this was not a traditional systematic review, we did use a standardized search strategy that could be replicated and have attempted to provide both a broad and thorough summary of the current literature. Many of the primary studies included within the review are limited by small sample sizes without comparison groups, in addition to the relative dearth of investigation from the settings where the bulk of HIV-infected children and adolescents live, particularly sub-Saharan Africa. We have tried to make clear these limitations when describing each study or content area, and to highlight the gaps in the existing research with recommendations for further investigation (Table 2). Our review emphasizes the need for mental health issues to be addressed proactively for all HIV-infected youth, and integrated into their overall HIV care.

**Table 2.** 

Key Areas for Future Mental Health Research and Practice Improvement for HIV-Infected Adolescents
Reliable and valid screening and assessment instruments of mental health that are culturally-appropriate for children and adolescents in resource-limited settings
Measurement instruments, mental health interventions, and mental health services for HIV-infected adolescents must be patient centered and adapted to specific cultural and environmental contexts
Longitudinal studies inclusive of perinatally infected and behaviourally infected youth with HIV to effectively monitor mental health outcomes across the lifespan
Evidence-based models linking the importance of mental health to treatment adherence and physical health outcomes for HIV populations
Integration of mental health services and interventions within adolescent-friendly healthcare services as youth with HIV transition to adult care
Incorporation of factors such as stigma and disclosure of illness to access to mental health services during the transition process

## List of abbreviations

ART, antiretroviral therapy; BDI, beck depression inventory; CES-D, center for epidemiological studies – depression scale; CDI, children’s depression inventory; HIV, human immunodeficiency virus; QoL, quality of life; RLS, resource-limited settings; RAD, reynold’s adolescent depression scale
